# Role of IDO expression in patients with locally advanced rectal cancer treated with preoperative chemoradiotherapy

**DOI:** 10.1186/s12885-022-10357-1

**Published:** 2022-12-05

**Authors:** Chie Takasu, Masaaki Nishi, Kozo Yoshikawa, Takuya Tokunaga, Toshihiro Nakao, Hideya Kashihara, Yuma Wada, Toshiaki Yoshimoto, Shohei Okikawa, Shoko Yamashita, Mitsuo Shimada

**Affiliations:** Department of Surgery, University of Tokushima, 3-18-15 Kuramoto-cho, Tokushima, 770-8503 Japan

**Keywords:** PD-L1, CRT, Immune escape, Neoadjuvant

## Abstract

**Background:**

The role of the immune system in locally advanced rectal cancer (LARC) following preoperative chemoradiotherapy (CRT) has been widely investigated in recent years. This study examined the prognostic significance of indoleamine-pyrrole 2,3-dioxygenase (IDO) expression in patients with LARC who received preoperative CRT.

**Methods:**

Ninety patients with LARC who underwent preoperative CRT and curative resection were enrolled. IDO and programmed death-ligand 1 (PD-L1) expression was evaluated by immunohistochemistry.

**Results:**

Clinicopathological factors did not significantly differ between patients with positive or negative IDO expression, excluding the correlation of positive IDO expression with better tumor differentiation (*p* = 0.02). IDO expression was not associated with pathological response (*p* = 0.44), but it was associated with PD-L1 expression. The 5-year overall survival (OS) rate was significantly worse in the IDO-positive group than in the IDO-negative group (64.8% vs. 85.4%, *p* = 0.02). Univariate analysis identified IDO and PD-L1 expression (*p* = 0.02), surgical procedure (*p* = 0.01), final pathological stage (*p* = 0.003), lymph node metastasis (*p* < 0.001), and lymphatic invasion (*p* = 0.002) as significant prognostic factors for OS. Multivariate analysis revealed that IDO expression (HR: 7.10, *p =* 0.0006), surgical procedure (HR: 5.03, *p =* 0.01), lymph node metastasis (HR: 2.37, *p =* 0.04) and lymphatic invasion (HR: 4.97, *p =* 0.01) were independent prognostic indicators. Disease-free survival was not correlated with IDO or PD-L1 expression.

**Conclusions:**

IDO expression in patients with LARC who received preoperative CRT could be a potential prognostic indicator. IDO expression could be a useful marker for specifying individual treatment strategies in LARC.

## Background

Chemoradiotherapy (CRT) is widely used as a standard therapy for locally advanced rectal cancer (LARC). Preoperative CRT can both reduce tumor size and the risk of local recurrence and increase the tumor resection rate. Furthermore, preoperative CRT helps preserve the anal sphincter and increase the anus retention rate, thereby maintaining patient quality of life [1].

Our group previously conducted a phase I study of preoperative CRT using tegafur/gimeracil/oteracil (S-1) plus oxaliplatin (SOX) and bevacizumab [2] following phase I and II studies of preoperative CRT using individual drugs [3]. We recently reported a phase II study of preoperative CRT using the SOX + bevacizumab regimen in patients with LARC [4]. The pathological response rates were not improved by the SOX + bevacizumab regimen (59%) compared with S-1 monotherapy (57%). This result indicated that the benefits of additional drugs during CRT are limited. Moreover, preoperative CRT might increase the risk of tumor growth in unresponsive patients. Our group also investigated potential predictive biomarkers such as surviving [5], microRNA-223 [6], and the neutrophil-to-lymphocyte ratio [7]. However, significant proportions of patients have poor responses to preoperative CRT. Despite the application of preoperative CRT, the improvement of overall survival (OS) and disease-free survival (DFS) has not been sufficient [8]. Better molecular markers, especially for discriminating prognosis in patients without pathological complete responses, are necessary for LARC.

The role of the immune system in colorectal cancer (CRC) has been widely investigated in recent years. We previously reported that programmed cell death protein 1 (PD-1) and programmed death-ligand 1 (PD-L1) expression was associated with poor prognosis in patients with CRC [9]. We also revealed the prognostic relevance of several immune-related molecules according to the sidedness of CRC tumors [10]. The prognostic factors were indoleamine-pyrrole 2,3-dioxygenase (IDO) in right-sided CRC and PD-L1 and forkhead box P3 (Foxp3) in left-sided CRC. The role of immune molecules in patients with LARC who received preoperative CRT has recently been illuminated. Although previous reports discussed the prognostic significance of PD-L1 [11–13], Foxp3, and tumor-infiltrating lymphocytes (TILs) [14], the prognostic impact of IDO in patients with LARC who received preoperative CRT remains unknown. Therefore, this study assessed the prognostic significance of IDO expression in patients with LARC who received preoperative CRT.

## Methods

### Patients

Ninety patients newly diagnosed with LARC who underwent CRT at the Department of Surgery in Tokushima University Hospital from 2008 to 2017 were enrolled in the present study. The study protocol was approved by the Tokushima University Hospital Institutional Ethics Committee (#1910), and informed consent was obtained from all participating patients.

CRT is routinely offered to patients with locally advanced cancers (≥T3 and/or node-positive) at the time of diagnosis and to those with distal T2N0 cancer near or involving the sphincter. CRT was performed as previously described [7]. Curative radical resection was performed 6–8 weeks after the completion of CRT. In total, ninety-three patients with LARC were registered in this study. One patient withdrew because of stroke. Two patients refused surgery after CRT because of good clinical responses. Demographic, patient and pathological variables were collected from clinical data available in the electronic medical record.

### Immunohistochemistry

Tissue samples for immunohistochemistry were fixed in formalin and embedded in paraffin. Samples were cut into 5-μm-thick serial sections, which were dewaxed, deparaffinized in xylene, and rehydrated using a series of decreasing alcohol concentrations. Samples were boiled in citrate buffer (pH 6.0) for 20 min in a microwave oven for antigen retrieval. The sections were incubated in Protein Block Serum-Free Reagent (DAKO, Carpinteria, CA, USA) for 30 min to block nonspecific binding. The slides were then incubated with primary antibodies overnight at 4 °C. The primary antibodies were a rabbit monoclonal antibody against PD-L1 (ab174838, 1:100; Abcam, Cambridge, UK) and a mouse monoclonal antibody against IDO (ab71276, 1:50; Abcam). Secondary antibody binding to these proteins was detected using an EnVision Dual Link System-HRP (K4065, Dako Corporation). A secondary peroxidase-labeled polymer conjugated to goat anti-mouse immunoglobulin was applied for 60 min. The sections were developed in 3,3-diaminobenzidine and counterstained with Mayer’s hematoxylin. Each slide was dehydrated using a series of increasing alcohol concentrations and then covered with a coverslip. Sections of human tonsils served as the positive control. The presence of positive cells on each slide was determined by a pathologist blinded to the origin of the samples.

PD-L1 and IDO expression was predominantly cytoplasmic, and the staining intensity was scored as follows: 0, no staining; 1+, weak staining; 2+, moderate staining; and 3+, strong staining. Distribution scores were determined by calculating the percentage of positive cancer cells and scoring the samples as follows: PD-L1, 0, 0–5%; 1+, 6–25%; 2+, 26–50%; 3+, 51–75%; and 4+, 76–100%; IDO, 0, 0%; 1+, 1–9%; 2+, 10–50%; 3+, 51–80%; and 4+, 81–100%. The total score was calculated as the sum of the staining intensity and distribution scores. PD-L1 positivity was indicated by a total score exceeding 3 [15, 16], whereas IDO positivity was indicated by a total score exceeding 4 [17].

### Statistical analysis

All statistical analyses were performed using JMP 8.0.1 (SAS, Cary, NC, USA). Continuous variables were compared using the Mann–Whitney U test, and categorical data were compared using the chi-squared test.

OS and DFS were calculated using the Kaplan–Meier method and compared using the log-rank test. Univariate and multivariate regression analyses of OS and DFS were performed using Cox’s proportional hazard model incorporating age at diagnosis (with 70 years as the cut point) and sex as patient factors, stage and main tumor location as pre-CRT factors, drug and surgical procedure as treatment factors, tumor differentiation, fStage, tumor depth, lymph node metastasis, venous invasion, lymphatic invasion, IDO expression, PD-L1 expression and pathological response as post-CRT factors to identify independent prognostic factors for OS and DFS. Statistically significant factors defined as *p* < 0.05 on univariate analysis were included in the multivariate regression analysis. Since final pathological stage and lymph node metastasis are confounders. We included one of these which showed stronger correlation in the multivariate regression analysis. Regarding the drug as a treatment factor, only one patient received 5-FU based chemotherapy. This patient was excluded for survival analysis.

## Results

The characteristics of the ninety patients are listed in Table 1. Median age was 65.8 years and 63 patients (70%) were men. Pre CRT stage was I (5.5%), II (31.1%) and III (63.3%). In more than 80% of the patients, the neoadjuvant CRT was performed with S-1.

**Table 1:** Patients’ clinicopathological characteristics

Variables	***n*** = 90
**< Patient’s factor >**	
Age (years)	65.9 ± 10.4
Sex (men/women)	63/27
**< Pre-CRT factor >**	
Stage (I/II/III)	5/28/57
Main tumor location (Ra/Rb/P)	12/75/3
**< Treatment factor >**	
Drug (S-1/UFT/5-FU)	73/16/1
Surgical procedure (LAR/ISR/APR/Local/TPE)	40/10/37/2/1
**< Post-CRT factor >**	
Tumor differentiation (diff./undiff.)	83/7
fStage (I/II/III)	28/31/31
T (1/2/3/4)	10/27/50/3
Lymph node metastasis (−/+)	62/28
Venous invasion (−/+)	45/45
Lymphatic invasion (−/+)	65/25
Pathological response grade (0/1/2)	1/57/32

*CRT* Chemoradiotherapy, *S-1* Tegafur/gimeracil/oteracil, *UFT* Tegafur/uracil, *5-FU* 5-fluorouracil, *diff./undiff*. differentiated histological type/undifferentiated histological type, *LAR* Low anterior resection, *ISR* Intersphincteric resection, *APR* Abdominoperineal resection, *Local* Local resection, *TPE* Total pelvic exenteration, *fStage* final pathological stage

### Immunohistochemistry of IDO and PD-L1

**Fig. 1:**
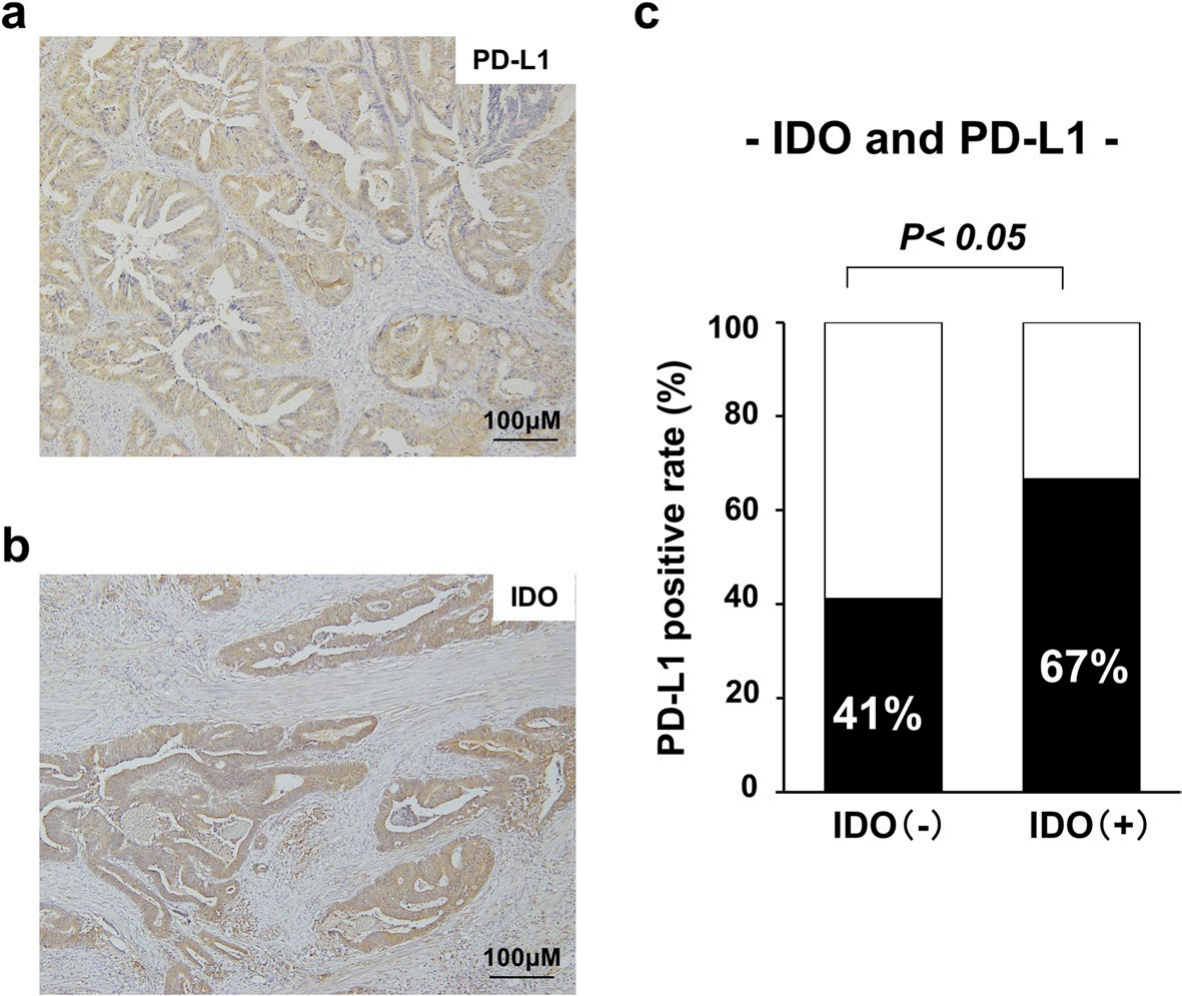
Indoleamine-pyrrole 2,3-dioxygenase (IDO) and programmed death-ligand 1 (PD-L1) expression in patients with locally advanced rectal cancer who received preoperative chemoradiotherapy. **a** IDO-positive expression in tumor cells (× 200). **b** PD-L1-positive expression in tumor cells (× 200). **c** Correlation between IDO and PD-L1 expression

Representative images of immunohistochemistry of IDO and PD-L1 were shown in Fig. 1 (IDO: Fig. 1a, PD-L1: Fig. 1b). Range of IDO and PD-L1 expression by intensity and distribution score was shown in Table 2. Regarding IDO expression, the average of intensity score, distribution score and total score were 1.05 ± 1.3, 0.89 ± 1.1 and 1.93 ± 2.4, respectively. Regarding PD-L1 expression, average of intensity score, distribution score and total score were 1.47 ± 1.0, 1.71 ± 1.2 and 3.2 ± 1.9, respectively.

**Table 2:** Immunohistochemistry results of IDO and PD-L1

Staining intensity	n (%)	Distribution	n (%)	Total score	n (%)
**< IDO expression>**					
**0**, no staining	52 (58%)	**0**, 0%	52 (58%)	**0**	52 (58%)
**1**+, weak staining	2 (2%)	**1**+, 1–9%	8 (9%)	**1**	0
**2**+, moderate staining	15 (17%)	**2**+, 10–50%	21 (23%)	**2**	0
**3**+, string staining	21 (23%)	**3**+, 51–80%	8 (9%)	**3**	8 (9%)
		**4**+, 81–100%	1 (1%)	**4**	3 (33%)
				**5**	25 (28%)
				**6**	1 (1%)
				**7**	1 (1%)
**< PD-L1 expression>**					
**0**, no staining	18 (20%)	**0**, 0–5%	18 (20%)	**0**	18 (20%)
**1**+, weak staining	23 (26%)	**1**+, 6–25%	16 (18%)	**1**	0
**2**+, moderate staining	38 (42%)	**2**+, 26–50%	38 (42%)	**2**	6 (7%)
**3**+, string staining	11 (12%)	**3**+, 51–75%	11 (12%)	**3**	20 (22%)
		**4**+, 76–100%	7 (8%)	**4**	25 (28%)
				**5**	14 (16%)
				**6**	5 (5%)
				**7**	2 (2%)

### Patient and tumor characteristics according to IDO expression

The characteristics of patients and tumors according to IDO expression are presented in Table 3. Following CRT, 30% of tumors were positive for IDO expression.

**Table 3:** Patients’ clinicopathological characteristics according to IDO expression

Variables	IDO (−) (***n*** = 63)	IDO (+) (***n*** = 27)	***p***-value
**< Patient’s factor >**			
Age (years)	66.3 ± 10.1	65.03 ± 11.1	0.62
Sex (men/women)	45/18	18 /9	0.65
**< Pre-CRT factor >**			
Stage (I,II/III)	21/42	12/15	0.31
Main tumor location (Ra/Rb,P)	10/53	2/25	0.26
**< Treatment factor >**			
Drug (S-1/UFT/5-FU)	54/9/0	19/7/1	0.11
Surgical procedure (LAR/ISR/APR/Local/TPE)	27/6/28/2/0	13/4/9/0/1	0.29
**< Post-CRT factor >**			
Tumor differentiation (diff./undiff.)	56/7	27/0	0.02
fStage (I, II/III)	39/24	20/7	0.26
T (1,2/3,4)	24/39	15/12	0.57
Lymph node metastasis (−/+)	42/21	20/7	0.48
Venous invasion (−/+)	35/28	10/17	0.11
Lymphatic invasion (−/+)	43/20	22/5	0.19
PD-L1 expression (−/+)	37/26	9/18	0.03
Pathological response (Non-responder /Responder)	39/24	19/8	0.44

*IDO* Indoleamine-pyrrole 2,3-dioxygenase, *Ra* Upper rectum, *Rb* Lower rectum, *P* anal canal, *S-1* tegafur/gimeracil/oteracil, *UFT* Tegafur/uracil, *5-FU* 5-fluorouracil, *diff./undiff*. differentiated histological type/undifferentiated histological type, *LAR* Low anterior resection, *LAR* Low anterior resection, *ISR* Intersphincteric resection, *APR* Abdominoperineal resection, *Local* local resection, *TPE* Total pelvic exenteration, *fStage* Final pathological stage

No significant difference was found in patient characteristics according to IDO expression. IDO expression was significantly correlated with greater tumor differentiation (*p* = 0.02). IDO expression tended to be correlated with venous invasion and lymphatic invasion (*p* = 0.11 and *p* = 0.19, respectively). Furthermore, the patient with IDO positive expression tended to have UFT regime than the patient with IDO negative expression (25.9% vs 14.3%, *p* = 0.11), although the differences were not statistically significant. There was no correlation between IDO expression and pathological response (*p* = 0.44). However, IDO expression was positively correlated with PD-L1 expression (*p* = 0.03, Fig. 1c).

### Associations of IDO expression on OS

**Fig. 2:**
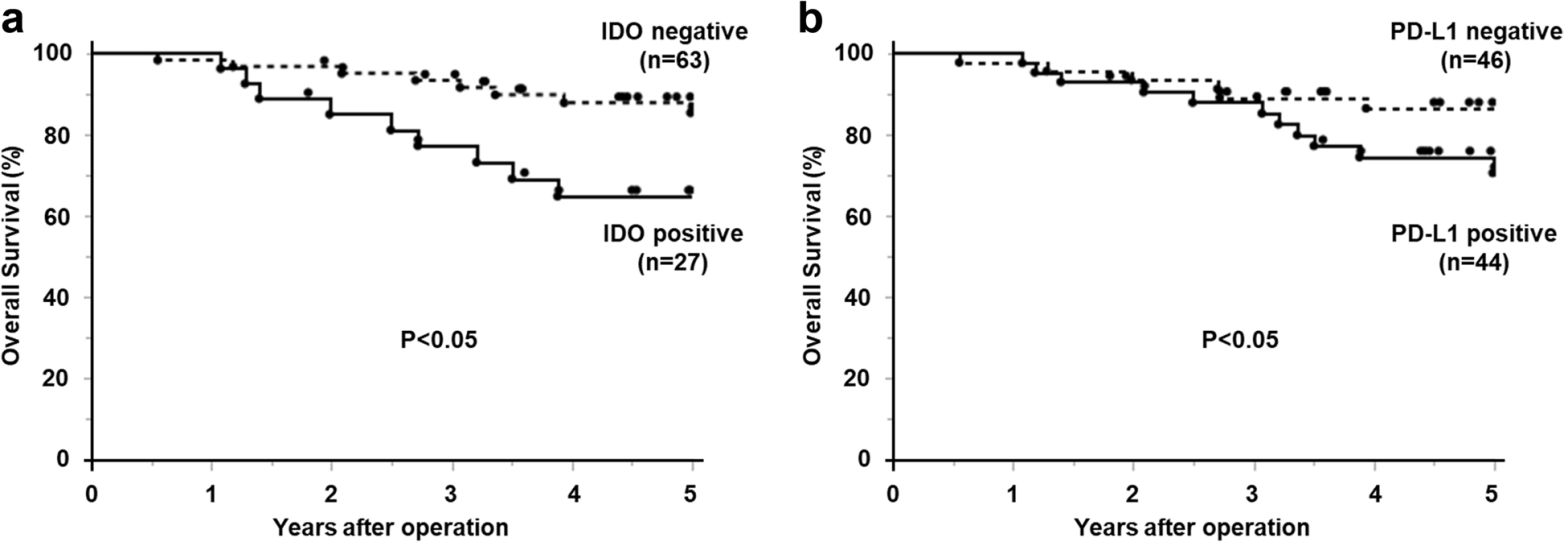
The OS rates according to IDO and PD-L1 expression. Kaplan–Meier analysis of 5-year OS for IDO and PD-L1 expression. **a** The 5-year OS rate was significantly worse in the IDO-positive group than in the IDO-negative group (64.8% vs. 85.4%). **b** The 5-year OS rate was significantly worse in the PD-L1–positive group than in the PD-L1–negative group (70.6% vs. 86.5%)

The 5-year OS rate was significantly worse in the IDO-positive group than in the IDO-negative group (64.8% vs. 85.4%, *p* = 0.02, Fig. 2a).

Univariate analysis identified IDO and PD-L1 expression (p = 0.02, Fig. 2b), surgical procedure (*p* = 0.01), final pathological stage (*p* = 0.003), lymph node metastasis (*p* < 0.001), and lymphatic invasion (*p* = 0.002) as significant prognostic factors for OS (Table 4). Regarding surgical procedure, the 5-year OS rate was 89.2% (low anterior resection, LAR), 76.2% (intersphincteric resection, ISR), 73.4% (abdominoperineal resection, APR), 50% (local resection) and 0% (total pelvic exenteration, TPE). For the survival analysis, surgical procedures were divided into two groups, LAR and the others (ISR, APR, local resection and TPE). Regarding drugs, the 5-year OS rate was 71.6% (S-1), 87.5% (UFT) and 0% (5-FU). Since there was only one patient who had 5-FU regime, the survival analysis was performed by two groups except 5-FU group.

**Table 4:** Univariate and multivariate analysis of clinicopathological factors associated with OS

Variables	5-year OS rate (%)	Univariate ***P***-value	Multivariate analysis	
			HR (95% CI)	***P***-value
**< Patient’s factor >**				
Age (< 70 years / ≥70 years)	79.8 / 78.5	0.56		
Sex (men/women)	84.4 / 65.4	0.08		
**< Pre-CRT factor >**				
Stage (I, II/III)	80.1 / 78.6	0.95		
Main tumor location (Ra/Rb, P)	83.3 / 78.6	0.80		
**< Treatment factor >**				
Drug (S-1/UFT)^a^	76.7 / 93.3	0.21		
Surgical procedure (LAR/ others)	89.2 / 70.9	0.01	5.03 (1.39–18.13)	0.04
**< Post-CRT factor >**				
Tumor differentiation (diff./undiff.)	78.6 /85.7	0.72		
fStage (I,II/III)**	89.4 / 56.6	0.003^b^		
T (1,2/3,4)	80.1 / /78.4	0.37		
Lymph node metastasis (−/+)	89.9 / 51.5	< 0.001	3.26 (1.05–10.10)	0.04
Venous invasion (−/+)	84.3 / 73.5	0.28		
Lymphatic invasion (−/+)	87.8 / 58.1	0.002	4.97 (1.37–18.04)	0.01
IDO (−: +)	85.4 / 64.8	0.02	7.10 (2.31–21.81)	0.0006
PD-L1 (−: +)	86.5 / 70.6	0.02	2.23 (0.81–6.12)	0.12
Pathological response (Non-responder /Responder)	89.5 / 73.5	0.03	1.28 (0.31–5.26)	0.73

*OS* Over-all survival, *CRT* Chemoradiotherapy, *Ra* Upper rectum, *Rb* Lower rectum, *P* Anal canal, *diff./undiff*. differentiated histological type/undifferentiated histological type, *IDO* Indoleamine-pyrrole 2,3-dioxygenase, *PD-L1* programmed death-ligand 1, *fStage* Final pathological stage, *HR* Hazard ratio, *CI* Confidence interval

^a^One patient who used 5-FU for CRT was excluded

^b^Final stage was excluded from multivariate analysis because it was confounder of lymph node metastasis

Multivariate analysis revealed that IDO expression (HR: 7.10, *p =* 0.0006), surgical procedure (HR: 5.03, *p =* 0.01), lymph node metastasis (HR: 2.37, *p =* 0.04) and lymphatic invasion (HR: 4.97, *p =* 0.01) were independent prognostic indicators.

### Associations of IDO expression on DFS

Regarding DFS, univariate analysis identified surgical procedure (*p* = 0.01), final pathological stage (*p* < 0.0001), lymph node metastasis (*p* = 0.0001), and lymphatic invasion (*p* < 0.0001) as significant prognostic factors (Table 5). The 5-year DFS rate was not correlated with IDO or PD-L1 expression (IDO: 69.0% vs. 62.6%, *p* = 0.53; PD-L1: 69.4% vs. 64.4%, *p* = 0.49). Regarding surgical procedure, the 5-year DFS rate was 79.8% (LAR), 58.3% (ISR), 57.9% (APR), 50% (local resection) and 0% (TPE). Regarding drugs, the 5-year DFS rate was 67.8% (S-1), 68.6% (UFT) and 0% (5-FU). Multivariate analysis revealed surgical procedure, LAR (*p* = 0.02) as a better independent risk factor for DFS (HR = 3.68, *p* = 0.02, Table 5).

**Table 5:** Univariate and multivariate analysis of clinicopathological factors associated with DFS

Variables	5-year DFS rate (%)	Univariate ***P***-value	Multivariate analysis	
			HR (95% CI)	***P***-value
**< Patient’s factor >**				
Age (< 70 years / ≥70 years)	64.3/71.2	0.35		
Sex (men/women)	70.7/58.6	0.24		
**< Pre-CRT factor >**				
Stage (I,II/III)	68.6/65.9	0.53		
Main tumor location (Ra/Rb, P)	83.3/64.5	0.28		
**< Treatment factor >**				
Drug (S-1/UFT)^a^	67.8 / 68.8	0.72		
Surgical procedure (LAR/ others)	79.8 / 56.5	0.03	2.95 (1.32–6.59	0.009
**< Post-CRT factor >**				
Tumor differentiation (diff./undiff.)	67.9/57.1	0.69		
fStage (I, II/III)	79.0/44.7	< 0.0001	2.64 (1.23–5.68)	0.01
T (1,2/3,4)	77.0/60.6	0.12		
Lymph node metastasis (−/+)^b^	78.3/42.9	0.0001		
Venous invasion (−/+)	70.8/63.1	0.24		
Lymphatic invasion (−/+)	77.6/40.0	< 0.0001	4.07 (1.86–8.91)	0.0005
IDO (−: +)	69.0/62.6	0.53		
PD-L1 (−: +)	69.4/64.4	0.49		
Pathological response (Non-responder /Responder)	74.5/62.8	0.13		

*DFS* Disease-free survival, *CRT* Chemoradiotherapy, *Ra* Upper rectum, *Rb* Lower rectum, *P* Anal canal, *diff./undiff*. Differentiated histological type/undifferentiated histological type, *IDO* Indoleamine-pyrrole 2,3-dioxygenase, *PD-L1* Programmed death-ligand 1, *fStage* Final pathological stage, *HR* Hazard ratio, *CI* Confidence interval

^a^One patient who used 5-FU for CRT was excluded

^b^Lymph node metastasis was excluded from multivariate analysis because it was confounder of fStage

Multivariate analysis revealed that surgical procedure (HR: 2.95, *p =* 0.009), fStage (HR: 2.64, *p =* 0.01) and lymphatic invasion (HR: 4.07, *p =* 0.0005) were independent prognostic indicators.

## Discussion

In this study, we revealed the impact of IDO expression in patients with LARC who received preoperative CRT. The IDO-positive group had significantly worse OS than the IDO-negative group. Furthermore, IDO was an independent prognostic factor that was positively correlated with PD-L1 expression.

In clinical settings, the selection of treatment after surgery with preoperative CRT is affected by the pathological response, final pathological stage, and physical conditions. Predictive prognostic markers using postoperative specimens from patients who received preoperative CRT could provide useful information to determine the indication of adjuvant chemotherapy. Recent research on the tumor microenvironment has focused on immune cells as well as the immunoescape system, including immune checkpoint molecules. These molecules, especially PD-L1, can be therapeutically targeted to produce significant clinical advantages in various tumors [18–20]. However, the prognostic role of the immunoescape system in rectal cancer, especially CRT, is not clearly understood.

We previously reported that PD-1 and PD-L1 expression was associated with poor prognosis in patients with CRC [9]. We also revealed the different prognostic relevance of several immune-related molecules according to the sidedness of CRC tumors [10]. Several reports have described PD-L1 expression in postoperative specimens from patients with LARC who received preoperative CRT, but the prognostic impact of PD-L1 is controversial [12, 21]. Only one report described the prognostic role of IDO in patients with LARC following pre-CRT. This report illustrated that patients with nodal-positive LARC and high IDO expression had better survival than those with low IDO expression, but the result was not significant. Conversely, the present study clearly demonstrated the significant prognostic relevance of IDO expression in LARC following pre-CRT.

IDO is an intracellular enzyme that catabolizes the conversion of tryptophan into kynurenine [22]. IDO is expressed in various types of human tumors. IDO arrests growth, activates cytotoxic T cells or natural killer cells [23], induces host regulatory T cells (Tregs) [24], and worsens survival [25]. Among cell types, lymphocytes are most sensitive to radiotherapy [26]. CRT might induce immune suppression because lymphocyte counts were significantly reduced by CRT [27]. The role of CD8+ cytotoxic T cells, which are most sensitive to radiation-induced apoptosis, in pre-CRT specimens is well established. The abundance of CD8+ TILs in pre-CRT specimens was associated with CRT sensitivity, resulting in favorable prognoses [14, 28, 29]. A previous report illustrated that high IDO-expressing tumors exhibited significantly lower numbers of TILs than IDO-negative tumors [25, 30]. This mechanism will be investigated in future research. Furthermore, we previously found that IDO expression in stage III gastric cancer is associated with poor prognosis and immunotolerance through the activation of Tregs [25]. CRT induces tumor apoptosis, which rapidly upregulates IDO expression [31]. IDO is an upstream signal for the induction of tolerogenic interleukin-10 and transforming growth factor-β, which recruit immunosuppressive Tregs [32]. In the present study, IDO was not associated with any clinicopathological feature other than PD-L1 expression. Both IDO and PD-L1 were prognostic for OS but not DFS. Furthermore, IDO was one of the independent prognostic factors for OS. Addition to IDO expression, surgical procedure (LAR) had an impact of survival for both OS and DFS. However, there was no correlation between IDO expression and surgical procedure. This indicates that IDO might affect both tumor characteristics and patients’ general characteristics such as vulnerability, frailty, and cachexia. Because the kynurenine pathway of tryptophan metabolism including IDO has received attention as a biomarker for the risk of frailty in the elderly, this relationship will be elucidated in a future study.

Some limitations of this study are worth mentioning. First, because this was a retrospective study based on data from one institute, there may be a potential risk of selection bias. In addition, the sample size was small. Furthermore, this study only used one analytical method (immunohistochemistry). Biomarker expression should be confirmed by determining mRNA levels in prospective studies.

## Conclusions

In conclusion, this study is the first to report the prognostic relevance of IDO in postoperative specimens from patients with LARC who received preoperative CRT. Applying IDO expression as a histological criterion will facilitate a more precise prediction of individual prognosis and therapeutic decisions in patients with LARC.

## Data Availability

The datasets analyzed during the current study are available from the corresponding author on reasonable request.

## Abbreviations

CRT: Chemoradiotherapy
LARC: Locally advanced rectal cancer
S-1: Tegafur/gimeracil/oteracil
OS: Overall survival
DFS: Disease-free survival
CRC: Colorectal cancer
PD-1: Programmed cell death protein 1
PD-L1: Programmed death-ligand 1
IDO: Indoleamine-pyrrole 2,3-dioxygenase
Foxp3: Forkhead box P3
TILs: Tumor-infiltrating lymphocytes
LAR: Low anterior resection
ISR: Intersphincteric resection
APR: Abdominoperineal resection
TPE: Total pelvic exenteration

## References

[1] Bosset JF, Collette L, Calais G, Mineur L, Maingon P, Radosevic-Jelic L, Daban A, Bardet E, Beny A, Ollier JC (2006). Chemotherapy with preoperative radiotherapy in rectal cancer. N Engl J Med.

[2] Sato H, Shimada M, Kurita N, Iwata T, Yoshikawa K, Higashigima J, Chikakio M, Kashihara H, Takasu C, Matsumoto N, Eto S (2015). Phase I trial of neoadjuvant preoperative chemotherapy with S-1, oxaliplatin, and bevacizumab plus radiation in patients with locally advanced rectal cancer. Int J Clin Oncol.

[3] Morimoto S, Shimada M, Kurita N, Sato H, Iwata T, Nishioka M, Yoshikawa K, Miyatani T, Kashihara H, Takasu C, Ikushima H (2012). Preoperative radiotherapy combined with S-1 for advanced lower rectal cancer: phase I trial. Hepato-gastroenterology.

[4] Higashijima J, Tokunaga T, Yoshimoto T, Eto S, Kashihara H, Takasu C, Nishi M, Yoshikawa K, Okitsu H, Ishikawa M, Miyake H, Yagi T, Kono T, Shimada M (2021). A multicenter phase II trial of preoperative chemoradiotherapy with S-1 plus oxaliplatin and bevacizumab for locally advanced rectal cancer. Int J Clin Oncol.

[5] Takasu C, Shimada M, Kurita N, Iwata T, Sato H, Nishioka M, Morimoto S, Yoshikawa K, Miyatani T, Kashihara H, Utsunomiya T, Uehara H (2013). Survivin expression can predict the effect of chemoradiotherapy for advanced lower rectal cancer. Int J Clin Oncol.

[6] Nakao T, Iwata T, Hotchi M, Yoshikawa K, Higashijima J, Nishi M, Takasu C, Eto S, Teraoku H, Shimada M (2015). Prediction of response to preoperative chemoradiotherapy and establishment of individualized therapy in advanced rectal cancer. Oncol Rep.

[7] Ishikawa D, Nishi M, Takasu C, Kashihara H, Tokunaga T, Higashijima J, Yoshikawa K, Shimada M (2020). The role of neutrophil-to-lymphocyte ratio on the effect of CRT for patients with rectal Cancer. In Vivo.

[8] Hong YS, Kim SY, Lee JS, Nam BH, Kim KP, Kim JE, Park YS, Park JO, Baek JY, Kim TY, Lee KW, Ahn JB, Lim SB, Yu CS, Kim JC, Yun SH, Kim JH, Park JH, Park HC, Jung KH, Kim TW (2019). Oxaliplatin-based adjuvant chemotherapy for rectal Cancer after preoperative Chemoradiotherapy (ADORE): long-term results of a randomized controlled trial. J Clin Oncol.

[9] Enkhbat T, Nishi M, Takasu C, Yoshikawa K, Jun H, Tokunaga T, Kashihara H, Ishikawa D, Shimada M (2018). Programmed cell death ligand 1 expression is an independent prognostic factor in colorectal Cancer. Anticancer Res.

[10] Takasu C, Nishi M, Yoshikawa K, Tokunaga T, Kashihara H, Yoshimoto T, Shimada M (2020). Impact of sidedness of colorectal cancer on tumor immunity. PLoS One.

[11] Hecht M, Buttner-Herold M, Erlenbach-Wunsch K, Haderlein M, Croner R, Grutzmann R, Hartmann A, Fietkau R, Distel LV (2016). PD-L1 is upregulated by radiochemotherapy in rectal adenocarcinoma patients and associated with a favourable prognosis. Eur J Cancer.

[12] Huemer F, Klieser E, Neureiter D, Schlintl V, Rinnerthaler G, Pages F, et al. Impact of PD-L1 scores and changes on clinical outcome in rectal Cancer patients undergoing neoadjuvant Chemoradiotherapy. J Clin Med. 2020;9(9). 10.3390/jcm9092775. 10.3390/jcm9092775 PMC7563312 32867256

[13] Lim YJ, Koh J, Kim S, Jeon SR, Chie EK, Kim K, Kang GH, Han SW, Kim TY, Jeong SY, Park KJ, Wu HG (2017). Chemoradiation-induced alteration of programmed death-ligand 1 and CD8(+) tumor-infiltrating lymphocytes identified patients with poor prognosis in rectal Cancer: a matched comparison analysis. Int J Radiat Oncol Biol Phys.

[14] Shinto E, Hase K, Hashiguchi Y, Sekizawa A, Ueno H, Shikina A, Kajiwara Y, Kobayashi H, Ishiguro M, Yamamoto J (2014). CD8+ and FOXP3+ tumor-infiltrating T cells before and after chemoradiotherapy for rectal cancer. Ann Surg Oncol.

[15] Eto S, Yoshikawa K, Nishi M, Higashijima J, Tokunaga T, Nakao T, Kashihara H, Takasu C, Iwata T, Shimada M (2016). Programmed cell death protein 1 expression is an independent prognostic factor in gastric cancer after curative resection. Gastric Cancer.

[16] Flies DB, Chen L (2007). The new B7s: playing a pivotal role in tumor immunity. J Immunother.

[17] Brandacher G, Perathoner A, Ladurner R, Schneeberger S, Obrist P, Winkler C, Werner ER, Werner-Felmayer G, Weiss HG, Gobel G, Margreiter R, Konigsrainer A, Fuchs D, Amberger A (2006). Prognostic value of indoleamine 2,3-dioxygenase expression in colorectal cancer: effect on tumor-infiltrating T cells. Clin Cancer Res.

[18] Rini BI, Plimack ER, Stus V, Gafanov R, Hawkins R, Nosov D, Pouliot F, Alekseev B, Soulieres D, Melichar B, Vynnychenko I, Kryzhanivska A, Bondarenko I, Azevedo SJ, Borchiellini D, Szczylik C, Markus M, McDermott RS, Bedke J, Tartas S, Chang YH, Tamada S, Shou Q, Perini RF, Chen M, Atkins MB, Powles T (2019). Pembrolizumab plus Axitinib versus Sunitinib for advanced renal-cell carcinoma. N Engl J Med.

[19] Schmid P, Adams S, Rugo HS, Schneeweiss A, Barrios CH, Iwata H, Dieras V, Hegg R, Im SA, Shaw Wright G, Henschel V, Molinero L, Chui SY, Funke R, Husain A, Winer EP, Loi S, Emens LA (2018). Atezolizumab and nab-paclitaxel in advanced triple-negative breast Cancer. N Engl J Med.

[20] Burtness B, Harrington KJ, Greil R, Soulieres D, Tahara M, de Castro G, Psyrri A, Baste N, Neupane P, Bratland A, Fuereder T, BGM H, Mesia R, Ngamphaiboon N, Rordorf T, Wan Ishak WZ, Hong RL, Gonzalez Mendoza R, Roy A, Zhang Y, Gumuscu B, Cheng JD, Jin F, Rischin D (2019). Pembrolizumab alone or with chemotherapy versus cetuximab with chemotherapy for recurrent or metastatic squamous cell carcinoma of the head and neck (KEYNOTE-048): a randomised, open-label, phase 3 study. Lancet.

[21] Saigusa S, Toiyama Y, Tanaka K, Inoue Y, Mori K, Ide S, Imaoka H, Kawamura M, Mohri Y, Kusunoki M (2016). Implication of programmed cell death ligand 1 expression in tumor recurrence and prognosis in rectal cancer with neoadjuvant chemoradiotherapy. Int J Clin Oncol.

[22] Ball HJ, Sanchez-Perez A, Weiser S, Austin CJ, Astelbauer F, Miu J, McQuillan JA, Stocker R, Jermiin LS, Hunt NH (2007). Characterization of an indoleamine 2,3-dioxygenase-like protein found in humans and mice. Gene.

[23] Munn DH, Sharma MD, Hou D, Baban B, Lee JR, Antonia SJ, Messina JL, Chandler P, Koni PA, Mellor AL (2004). Expression of indoleamine 2,3-dioxygenase by plasmacytoid dendritic cells in tumor-draining lymph nodes. J Clin Invest.

[24] Wainwright DA, Balyasnikova IV, Chang AL, Ahmed AU, Moon KS, Auffinger B, Tobias AL, Han Y, Lesniak MS (2012). IDO expression in brain tumors increases the recruitment of regulatory T cells and negatively impacts survival. Clin Cancer Res.

[25] Godin-Ethier J, Hanafi LA, Piccirillo CA, Lapointe R (2011). Indoleamine 2,3-dioxygenase expression in human cancers: clinical and immunologic perspectives. Clin Cancer Res.

[26] Stewart CC, Perez CA (1976). Effect of irradiation on immune responses. Radiology.

[27] Kitayama J, Yasuda K, Kawai K, Sunami E, Nagawa H (2010). Circulating lymphocyte number has a positive association with tumor response in neoadjuvant chemoradiotherapy for advanced rectal cancer. Radiat Oncol.

[28] Tsuchikawa T, Hirano S, Tanaka E, Matsumoto J, Kato K, Nakamura T, Ebihara Y, Shichinohe T (2013). Novel aspects of preoperative chemoradiation therapy improving anti-tumor immunity in pancreatic cancer. Cancer Sci.

[29] Yasuda K, Nirei T, Sunami E, Nagawa H, Kitayama J (2011). Density of CD4(+) and CD8(+) T lymphocytes in biopsy samples can be a predictor of pathological response to chemoradiotherapy (CRT) for rectal cancer. Radiat Oncol.

[30] Han Y, Yang Y, Chen Z, Jiang Z, Gu Y, Liu Y, Xu S, Lin C, Pan Z, Zhou W, Cao X (2014). Human hepatocellular carcinoma-infiltrating CD4(+)CD69(+)Foxp3(−) regulatory T cell suppresses T cell response via membrane-bound TGF-beta1. J Mol Med (Berl).

[31] Ravishankar B, Liu H, Shinde R, Chandler P, Baban B, Tanaka M, Munn DH, Mellor AL, Karlsson MC, McGaha TL (2012). Tolerance to apoptotic cells is regulated by indoleamine 2,3-dioxygenase. Proc Natl Acad Sci U S A.

[32] Ravishankar B, Shinde R, Liu H, Chaudhary K, Bradley J, Lemos HP, Chandler P, Tanaka M, Munn DH, Mellor AL, McGaha TL (2014). Marginal zone CD169+ macrophages coordinate apoptotic cell-driven cellular recruitment and tolerance. Proc Natl Acad Sci U S A.

